# Disparities in overall and site-specific cancer mortality among immigrant generations in Sweden: a nationwide follow-up study over 3 decades

**DOI:** 10.1093/aje/kwae388

**Published:** 2024-10-04

**Authors:** Daniel Nigusse Tollosa, Kazem Zendehdel, Paolo Boffetta, Eero Pukkala, Mikael Rostila

**Affiliations:** Department of Public Health Sciences, Stockholm University, Stockholm, Sweden; Cancer Research Center, Cancer Institute, Tehran University of Medical Sciences, Tehran, Iran; Department of Medical and Surgical Sciences, University of Bologna, Bologna, Italy; Department of Medical and Surgical Sciences, University of Bologna, Bologna, Italy; Stony Brook Cancer Center, Stony Brook University, Stony Brook, NY, United States; Finnish Cancer Registry, Institute for Statistical and Epidemiological Cancer Research, Helsinki, Finland; Health Sciences Unit, Faculty of Social Sciences, Tampere University, Tampere, Finland; Department of Public Health Sciences, Stockholm University, Stockholm, Sweden; Centre for Health Equity Studies, Stockholm University/Karolinska Institutet, Stockholm, Sweden; Aging Research Center, Karolinska Institutet, Stockholm, Sweden

**Keywords:** cancer, mortality, immigrant generations, registry-based, Sweden

## Abstract

We examined the overall and site-specific cancer mortality disparities among first-generation—separately in adults (G1) and children (G1.5) at immigration—and second-generation (G2) immigrants and their countries of origin, using population-based registries in Sweden, encompassing more than 8.5 million individuals aged 20 years or older residing in Sweden since 1990, with follow-up until December 31, 2023. Cox proportional hazard models were fitted, stratified by sex, to estimate hazard ratios and 95% CIs compared with native Swedes. Mortality rates for most cancers transitioned from lower in G1 toward the rate of natives in G2. However, elevated mortality rates were sustained across generations for liver cancer in men and stomach cancer in women. Among G2, mortality rates associated with lymphohematopoietic cancers in men and lung and cervix uteri cancers in women were elevated y 10%, 9%, and 17%, respectively, compared to native Swedes. Country of origin analyses revealed substantial disparities. For instance, G2 women with Nordic parental origin had a 13% higher risk of death from lung cancer, whereas those with non-Western parental origin had a 54% lower risk, as compared to native Swedes. These findings suggest generational and arrival-age dynamics of cancer mortality and highlight target groups for cancer prevention and control among immigrants.

## Introduction

Sweden has a long history of immigration, with a significant increase in migration since the end of World War II that has reshaped the demographic landscape of the country. This trajectory is expected to continue.^1^^,^^2^ Consequently, the descendants of immigrants—the so-called second-generation immigrants and children who arrived at a young age—are growing in proportion and diversity, and in the proportion of age categories in which cancer incidence and mortality rates are high. These trends result in a demand for an emphasis on health studies among these population segments to improve the health care response. Second-generation immigrants were estimated to account for approximately 14% of the total population in 2023,^3^ and the percentage of live births to immigrant mothers has increased from 18% in 2000 to 31% in 2021 in Sweden.^4^

Although descendants of immigrants had elevated all-cause mortality rates compared with the native population in several studies,^5–7^ the overall cancer mortality rate often resembles that of native individuals in Sweden^8^^,^^9^ and other countries.^10^^,^^11^ This mortality trend is often explained by “the health convergency” concept, referring to the mortality advantage observed in first-generation immigrants^12–15^—also known as the “healthy migrant effect”—whereby mortality rates tend to decline over time and with the subsequent generations.^16^ Given the diverse nature of cancer types, each with distinct etiologies and progressions, the observed resemblances in all-site cancer mortality rate with the native population may obfuscate cancer-specific differences. However, detailed analyses of mortality disparities by specific cancer types are limited. Furthermore, cancer mortality rates of young immigrants at arrival are barely assessed, despite some studies reporting elevated overall mortality in this group compared with the native population.^17^ Immigrants who arrive at a young age tend to have a longer potential exposure to the host country’s environment and undergo acculturation at a faster pace than those who migrate later in life.

Immigrant parents often pass on genetic predisposition, cultural behaviors, and lifestyle choices to their children. This may contribute to differences in cancer mortality rates among second-generation immigrants, especially when socioeconomic inequalities extend from the first-generation immigrants to their offspring. For instance, Rooth et al.^18^ noted that children of immigrants, particularly those having a non-European background and, to some extent, those with a South European background, had lower employment rates and incomes than natives in Sweden. The impact of socioeconomic status on cancer mortality disparities is well documented in many studies.^19^^,^^20^

Previous studies examining cancer mortality disparities among immigrants in Sweden have been limited, focusing either on all-site cancers or only on first-generation immigrants, and they are outdated and lacked statistical power. We explored disparities in cancer mortality rates—all sites combined and 13 specific cancers—by immigrant generations and their countries of origin in Sweden, using cancer mortality data from between 1990 and 2023.

## Methods

### Cohort and data sources

**Figure 1:**
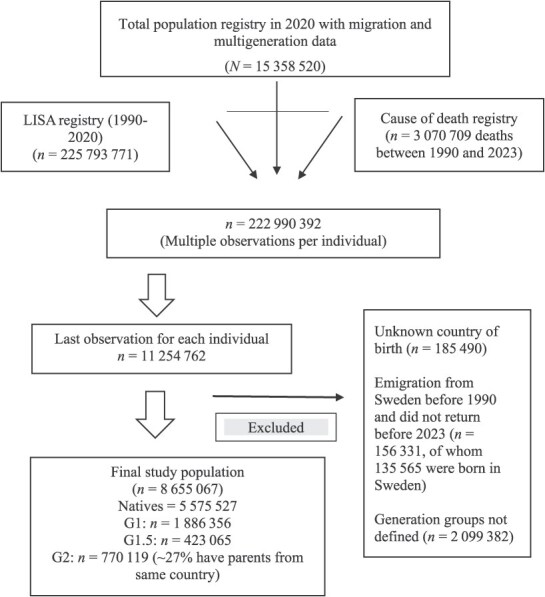
Final study population and exclusion criteria, Sweden, 1990-2023. The Longitudinal Database of Health, Insurance, and Labor Market Studies (LISA) includes multiple observations of individuals aged ≥20 years. Excluded multiple observations at age <20 years (*n* = 14 907 924). G1, adult (arrived when aged ≥18 years) first-generation immigrants; G1.5, child (arrived when aged <18 years) first-generation immigrants; G2, children of immigrants born in Sweden (second-generation immigrants).

We conducted a population-based cohort study, including adult individuals aged 20 years or older who had been residing in Sweden since January 1, 1990, and followed them until they emigrated, died, or reached the end of the study on December 31, 2023, whichever came first. Multiple Swedish registry data sources were used and merged using a pseudonymized personal identification number. Information about the participant’s background (specifically, the country of birth), date of birth, sex, and in- and out-migration data were obtained from the Total Population Registry.^21^ The Multi-Generation Registry was used to extract the links of individuals with their biological parents.^22^ Information about the sociodemographic characteristics (eg, education, disposable income, and civil status) was taken from the Longitudinal Database of Health, Insurance, and Labor Market Studies, which collects annual data covering the Swedish population aged ≥16 years registered since 1990 (individuals aged 15 years added since 2010).^23^ The Cause of Death Registry was used to retrieve information on the date and underlying causes of death, completed with the corresponding *International Classification of Diseases*, Ninth Revision (*ICD-9*; from 1990 to 1996) and *ICD-10* (from 1997 onward) codes.^24^  Figure 1 shows the selection of the study population and exclusion criteria.

The study was approved by the Swedish Ethical Review Authority (decisions 2017/716-31/5 and 2023-02550-02).

### Variables

Background information of the individuals (ie, either born in Sweden or another country and age at immigration), specifically for those born outside Sweden, was used to categorize them into 1) people who were born in Sweden (natives) along with their parents; 2) adult first-generation immigrants (G1), that is, foreign-born individuals who migrated to Sweden at age 18 years or older; 3) child first-generation immigrants (G1.5), that is, foreign-born individuals who migrated to Sweden before the age of 18 years; and 4) descendants of immigrants (second generation [G2]), those born in Sweden with at least 1 foreign-born parent. We further grouped immigrant generations into the following regions and countries based on their country of birth for G1 and G1.5, and parents’ birth countries for G2: Nordic (Finland, Denmark, Norway); all Europe excluding Nordic (ie, Central and Eastern Europe include Poland, former Yugoslavia, former Czechoslovakia, Estonia, other former Soviet Union countries excluding Estonia, and the rest of Europe); non-Western regions (South America, the Middle East, Asia, and Africa); and rest of the world. Analyses were also performed for specific countries with a reasonable number of cancer deaths across the generation groups. The following criteria were used to determine the country of origin for G2 individuals: 1) if parents came from different regions/countries, priority was given to the non-Western origin/country, or 2) the mother’s country of birth was used for those who had a different parental country of birth but came from the same region. However, if the mother was born in Sweden, we used the father’s country of birth.

The primary outcome of this study was death due to cancer, all cancers combined (*ICD-9*: 140-239; *ICD-10*: C00-C99) and specifically for lung (*ICD-9*: 162; *ICD-10*: C33-C34), colorectal (*ICD-9*: 153-154; *ICD-10*: C18-C21), pancreas (*ICD-9*: 157; *ICD-10*: C25), liver (*ICD-9*: 155; *ICD-10*: C22), stomach (*ICD-9*: 151; *ICD-10*: C16), brain and central nervous system (CNS) (*ICD-9*: 191-192; *ICD-10*: C70-C72), lymphohematopoietic (*ICD-9*: 200-208; *ICD-10*: C81-C85 and C90-C96), melanoma (*ICD-9*: 172; *ICD-10*: C43), and sex-specific cancers including prostate (*ICD-9*: 185; *ICD-10*: C61) in men, and breast (*ICD-9*: 174; *ICD-10*: C50), cervix uteri (*ICD-9*: 180, *ICD-10*: C53), corpus uteri (*ICD-9*: 182, *ICD-10*: C54), and ovary (*ICD-9*: 183, *ICD-10*: C56) in women. Owing to a small number of events for other cancers, especially in G1.5 and G2 groups, we restricted our analysis by country of origin for cancers of the lung, colorectal, female breast, prostate, cervix uteri, stomach, and male lymphohematopoietic cancers.

Covariates in this study were attained age, education level (classified into primary school, secondary school, college/university, or unknown or missing data); individuals’ disposable income (divided into quintile groups: very low, low, medium, high, and very high), and marital status (single, married, divorced, or widowed). All these characteristics were observed at the exit date of the study. Estimates were also adjusted for calendar period split into 5-year intervals from 1990-1994 to 2015-2019, and 2020-2023.

### Statistical methods

Cox proportional hazard regression models with age as the underlying time scale were fitted to assess the relative risk of all-cancer and cancer-specific mortality rates and represented as hazard ratios (HRs) with corresponding 95% CIs. The HRs were estimated by generation groups (G1, G1.5, and G2) and country of origin (forest plots were used to visualize the HRs), using native individuals as a reference group. Two multivariable Cox regression models were fitted: a simple model, adjusting for age and calendar year, and a fully adjusted model, adjusting for age, calendar year, disposable income, education, and marital status. All analyses were stratified by sex. The Akaike information criterion and Bayesian information criterion were used for model selection, suggesting that the model with all covariates best fits the data in all regression models. Stata software (version 17; Stata Corp) was used for analyses.

## Results

### Characteristics of the study population

The study included 8 655 067 individuals, accumulating 195 million person-years and 308 098 cancer deaths. The immigrant generations (G1, G1.5, and G2) comprised 20%, 5%, and 9% of the total study population, respectively. The distribution of disposable income and educational status is comparable between native Swedes and G2 individuals; however, relative to the other generation groups, a low proportion of G1.5 individuals completed tertiary education, and a high proportion of G1 and G1.5 individuals were in the lowest quantile group than the native individuals. This difference was more notable in women (Table 1).

**Table 1:** Person-years and number of all-site cancer deaths across the characteristics of the study population at the end of their follow-up, stratified by sex and immigrant generation groups in Sweden, 1990-2023.

**Characteristic** ^**a**^	**Percentage/100 000 person-years: men (no. of deaths)**				**Percentage/100 000 person-years: women (no. of deaths)**			
	**Native (*n* = 2 859 125)**	**G1** ^**b**^ **(*n* = 955 942)**	**G1.5** ^**c**^ **(*n* = 219 339)**	**G2** ^**d**^ **(*n* = 395 622)**	**Natives (n = 2 716 402)**	**G1** ^**b**^ **(*n* = 930 414)**	**G1.5** ^**c**^ **(*n* = 203 726)**	**G2** ^**d**^ **(*n* = 374 497)**
Overall	100/721.7 (115 654)	100/134.9 (33 009)	100/41.2 (3264)	100/84.9 (6245)	100/696.2 (108 174)	100/146.7 (31 971)	100/40.6 (3656)	100/80.4 (6125)
Age, years								
20-39	41.8/215.2 (5858)	40.4/26.9 (382)	60.2/15.1 (201)	61.3/38.1 (829)	29.3/102.5 (2919)	49.6/51.3 (1581)	75.1/24.6 (666)	48.4/21.4 (424)
40-59	32.8/280.1 (45 227)	33.2/48.5 (5015)	57.9/16.9 (932)	28.0/33.3 (2862)	32.1/256.7 (29 475)	30.1/55.1 (9, 557)	18.4/11.8 (1833)	33.7/37.0 (2534)
≥60	25.3/226.5 (64 465)	26.4/59.4 (27 042)	13.4/9.3 (2121)	10.7/13.5 (2518)	38.6/336.9 (75, 672)	20.3/40.3 (2 0293)	6.5/4.0 (1155)	17.9/21.9 (3146)
Disposable income quintile								
1 (lowest)	14.4/83.2 (17 720)	35.3/31.6 (11 538)	23.8/6.3 (489)	23.5/14.8 (1237)	10.0/52.8 (25 238)	55.4/65.7 (19 938)	39.1/13.1 (1375)	14.7 (7.5) (1120)
2	19.2/139.5 (42 631)	16.7/27.8 (12 300)	14.3/5.7 (1158)	16.8/14.3 (2012)	20.5/147.6 (44 665)	19.8/38.3 (8291)	25.7/11.5 (1461)	15.6 (12.1) (2270)
3	20.7/154.6 (30 439)	15.7/25.5 (5358)	15.9/6.6 (885)	17.0/14.7 (1392)	22.3/153.3 (22 668)	13.0/23.1 (2227)	19.3/8.4 (557)	19.8 (15.3) (1426)
4	20.9/149.6 (13 654)	16.5/24.5 (1885)	21.8/9.7 (435)	19.7/17.5 (802)	24.9/171.8 (9258)	7.5/12.8 (642)	10.9/4.9 (184)	26.2 (22.4) (785)
5 (highest)	24.7/194.8 (11 106)	15.8/25.3 (1, 358)	24.2/12.9 (287)	23.0/23.5 (766)	22.5/170.7 (6237)	4.2/6.8 (333)	5.1/2.6 (77)	23.7 (23.1) (503)
Education status								
Primary completed	18.3/141.0 (44 278)	20.6/32.7 (12 181)	22.1/8.8 (1203)	15.3/13.2 (1792)	13.1/99.4 (34 672)	23.5/41.4 (13 059)	20.9/8.6 (1332)	9.6/8.1 (1343)
Secondary completed	50.7/356.8 (47 950)	27.4/47.2 (11 838)	48.7/20.5 (1523)	52.0/43.7 (2917)	43.2/303.4 (47 629)	23.6/44.3 (9757)	47.6/19.7 (1668)	43.4/35.3 (2970)
Tertiary completed	30.4/221.1 (22 855)	34.0/44.5 (5257)	25.1/11.2 (510)	31.1/27.2 (1445)	43.3/291.7 (25 495)	33.2/45.5 (4559)	26.9/11.4 (636)	45.9/36.5 (1767)
Unknown or missing data	0.6/2.9 (467)	18.1/10.4 (3163)	4.1/0.7 (18)	1.5/0.7 (55)	0.4/1.7 (270)	19.8/15.6 (4056)	4.5/0.8 (18)	1.1/0.4 (24)
Marital status								
Single	50.6/301.4 (24 124)	31.8/27.4 (3214)	60.7/19.0 (829)	66.2/46.8 (2015)	37.9/198.4 (16 692)	23.6/22.9 (2465)	58.3/18.3 (730)	51.2/31.1 (1470)
Married	38.3/324.9 (67 037)	50.5/73.5 (19041)	28.5/15.8 (1601)	26.1/29.1 (2941)	41.7/325.8 (53 428)	54.6/81.6 (15374)	30.3/33.4 (1781)	34.2/33.4 (2736)
Divorced	9.6/80.2 (19 564)	15.1/28.3 (6799)	9.9/8.3 (656)	7.2/8.3 (1075)	13.5/110.5 (21 526)	14.7/28.7 (6560)	10.1/5.7) (875)	12.3/13.1 (1339)
Widowed	1.6/13.9 (4151)	2.6/5.1 (3039)	0.8/0.5 (139)	0.5/0.6 (159)	6.8/58.8 (13878)	7.1/12.1 (6628)	1.2/0.7) (247)	2.2/2.6 (480)
Calendar year								
1990-1994	11.5/82.3 (3346)	10.1/12.8 (3333)	0.95/3.0 (31)	3.2/7.6 (195)	3.7/78.2 (3948)	10.2/1.4 (3265)	0.6/3.2 (21)	3.3/7.1 (199)
1995-1999	12.6/93.9 (6810)	11.9/14.1 (3916)	3.8/3.9 (124)	5.3/9.3 (330)	7.0/89.4 (7613)	12.6/15.9 (4024)	2.9/4.1 (142)	5.6/8.7 (344)
2000-2004	13.6/101.4 (10 832)	13.3/15.2 (4373)	10.7/4.8 (349)	7.7/10.8 (479)	10.3/96.9 (11 157)	13.7/17.4 (4387)	10.7/4.9 (392)	9.4/10.1 (575)
2005-2009	14.6/108.7 (16 572)	15.4/17.8 (5090)	14.8/6.0 (484)	13.4/12.4 (838)	14.6/104.3 (15 766)	15.5/20.1 (4965)	15.9/5.9 (582)	13.5/11.6 (827)
2010-2014	15.7/116.6 (23 112)	17.4/22.1 (5729)	20.2/7.1 (658)	18.4/14.4 (1146)	19.4/112.7 (21 031)	16.4/24.3 (5236)	19.9/6.9 (731)	19.1/11.6 (1172)
2015-2019	16.3/121.7 (30 288)	18.6/27.9 (6145)	27.2/8.5 (887)	26.8/16.4 (1675)	24.8/118.8 (26 826)	18.4/9.3 (5876)	26.7/8.2 (977)	26.1/15.7 (1600)
2020-2023	15.8/97.1 (24 694)	13.4/24.8 (4423)	22.4/7.7 (731)	25.3/13.9 (1582)	20.1/95.6 (21 833)	13.2/25.5 (4218)	22.2/7.2 (811)	22.9/13.3 (1408)

^a^ All characteristics were observed at the exit date of the study.

^b^ G1 refers to adult (arrived when aged ≥18 years) first-generation immigrants.

^c^ G1.5 refers to child (arrived when aged <18 years) first-generation immigrants.

^d^ G2 refers to children of immigrants born in Sweden (second-generation immigrants).

### All-site and specific-cancer mortality by immigrant generations

Mortality rates of G1 immigrants generally were either significantly lower or not notable differences in mortality rates for most cancer types compared with native Swedes. However, elevated mortality rates were observed in men for all-site cancers combined (HR = 1.07; 95% CI, 1.06-1.09), lung (HR = 1.71; 95% CI, 1.65-1.76), stomach (HR = 1.72; 95% CI, 1.61-1.84), and liver (HR = 1.39; 95% CI, 1.30-1.49), and in women for stomach cancer (HR = 1.61; 95% CI, 1.47-1.76) (Table 2).

**Table 2:** Hazard ratios and 95% CIs of cancer deaths by immigrant generations compared with native Swedes, overall and by cancer type in Sweden, 1990-2023.

**Cancer type**	**Generation group** ^**a**^	**Male immigrants**			**Female immigrants**		
		**No. of cases**	**HR (95% CI)** ^**b**^	**HR (95% CI)** ^**c**^	**No. of cases**	**HR (95% CI)** ^**b**^	**HR (95% CI)** ^**c**^
All sites	G1	33 009	1.08 (1.06-1.09)	1.07 (1.06-1.09)	31 971	0.93 (0.91-0.94)	0.86 (0.84-0.87)
	G1.5	3264	1.11 (1.07-1.15)	1.10 (1.06-1.14)	3656	1.03 (0.99-1.06)	0.95 (0.92-0.98)
	G2	6245	1.04 (1.01-1.07)	1.04 (1.02-1.07)	6125	1.02 (0.99-1.04)	1.02 (0.99-1.05)
Lung	G1	8252	1.74 (1.68-1.79)	1.71 (1.65-1.76)	5140	0.89 (0.86-0.93)	0.82 (0.79-0.86)
	G1.5	617	1.36 (1.25-1.47)	1.32 (1.22-1.43)	730	1.15 (1.07-1.24)	1.03 (0.96-1.11)
	G2	953	1.04 (0.97-1.11)	1.06 (0.99-1.13)	1141	1.08 (1.02-1.15)	1.09 (1.02-1.16)
Prostate	G1	4386	0.77 (0.74-0.81)	0.78 (0.74-0.82)	NA	NA	NA
	G1.5	331	0.99 (0.89-1.12)	0.99 (0.89-1.12)	NA	NA	NA
	G2	598	1.01 (0.93-1.09)	1.02 (0.94-1.11)	NA	NA	NA
Breast	G1	NA	NA	NA	4587	0.97 (0.93-1.00)	0.91 (0.87-0.94)
	G1.5	NA	NA	NA	560	0.94 (0.86-1.03)	0.89 (0.82-0.97)
	G2	NA	NA	NA	1148	1.05 (0.98-1.11)	1.05 (0.99-1.12)
Colorectal	G1	3446	0.90 (0.86-0.94)	0.91 (0.87-0.95)	3599	0.84 (0.80-0.88)	0.79 (0.75-0.83)
	G1.5	381	1.06 (0.95-1.16)	1.06 (0.95-1.17)	371	0.98 (0.88-1.08)	0.91 (0.81-1.01)
	G2	748	0.99 (0.91-1.06)	0.99 (0.92-1.06)	565	0.91 (0.83-0.99)	0.92 (0.84-1.00)
Stomach	G1	1752	1.72 (1.60-1.84)	1.72 (1.61-1.84)	1324	1.81 (1.66-1.97)	1.61 (1.47-1.76)
	G1.5	172	1.71 (1.46-2.00)	1.70 (1.45-1.99)	148	2.04 (1.72-2.41)	1.84 (1.55-2.18)
	G2	217	1.07 (0.93-1.22)	1.09 (0.95-1.25)	157	1.25 (1.06-1.48)	1.26 (1.07-1.49)
Liver	G1	1503	1.41 (1.32-1.51)	1.39 (1.30-1.49)	888	1.15 (1.04-1.27)	1.02 (0.91-1.13)
	G1.5	168	1.37 (1.17-1.60)	1.34 (1.14-1.56)	90	1.28 (1.03-1.58)	1.16 (0.94-1.44)
	G2	342	1.30 (1.16-1.45)	1.30 (1.16-1.45)	110	0.94 (0.77-1.14)	0.93 (0.76-1.13)
Pancreas	G1	2270	0.99 (0.94-1.04)	0.99 (0.95-1.05)	2536	0.90 (0.86-0.96)	0.85 (0.81-0.90)
	G1.5	268	1.07 (0.95-1.21)	1.06 (0.93-1.20)	319	1.15 (1.03-1.29)	1.07 (0.95-1.20)
	G2	536	1.03 (0.94-1.12)	1.03 (0.94-1.12)	437	0.97 (0.88-1.06)	0.98 (0.88-1.08)
Melanoma	G1	470	0.51 (0.45-0.56)	0.51 (0.45-0.57)	386	0.48 (0.42-0.55)	0.44 (0.38-0.51)
	G1.5	74	0.64 (0.50-0.80)	0.65 (0.52-0.83)	53	0.58 (0.44-0.77)	0.55 (0.42-0.72)
	G2	227	0.92 (0.80-1.05)	0.91 (0.79-1.04)	168	0.99 (0.85-1.17)	1.00 (0.85-1.17)
Brain and central nervous system	G1	844	0.68 (0.62-0.73)	0.69 (0.64-0.76)	743	0.78 (0.71-0.85)	0.71 (0.65-0.78)
	G1.5	154	0.80 (0.68-0.94)	0.81 (0.69-0.95)	111	0.77 (0.64-0.93)	0.73 (0.61-0.89)
	G2	428	0.99 (0.89-1.09)	0.98 (0.88-1.08)	239	0.88 (0.77-1.01)	0.88 (0.77-1.01)
Lymphohematopoietic	G1	2802	0.99 (0.94-1.04)	0.99 (0.94-1.05)	2667	1.02 (0.96-1.07)	0.94 (0.88-1.00)
	G1.5	309	1.09 (0.97-1.22)	1.10 (0.98-1.23)	252	1.07 (0.94-1.21)	0.99 (0.88-1.13)
	G2	635	1.10 (1.01-1.19)	1.08 (1.00-1.18)	368	0.99 (0.89-1.10)	0.99 (0.88-1.10)
Cervix uteri	G1	NA	NA	NA	584	1.13 (1.02-1.26)	1.01 (0.89-1.13)
	G1.5	NA	NA	NA	74	0.98 (0.77-1.24)	0.85 (0.67-1.07)
	G2	NA	NA	NA	174	1.21 (1.03-1.42)	1.17 (1.01-1.37)
Corpus uteri	G1	NA	NA	NA	1013	1.03 (0.94-1.12)	0.99 (0.91-1.08)
	G1.5	NA	NA	NA	86	0.89 (0.72-1.04)	0.83 (0.67-1.03)
	G2	NA	NA	NA	175	1.10 (0.95-1.29)	1.12 (0.96-1.31)
Ovary	G1	NA	NA	NA	1802	0.91 (0.85-0.96)	0.87 (0.82-0.93)
	G1.5	NA	NA	NA	214	0.90 (0.78-1.03)	0.84 (0.73-0.97)
	G2	NA	NA	NA	418	1.03 (0.93-1.13)	1.04 (0.94-1.15)

Abbreviations: HR, hazard ratio; NA, not applicable.

^a^G1 refers to adult (arrived when aged ≥18 years) first-generation immigrants. G1.5 refers to child (arrived when aged <18 years) first-generation immigrants. G2 refers to children of immigrants born in Sweden (second-generation immigrants).

^b^HRs were adjusted for age and calendar year.

^c^HRs were adjusted for age, educational status, disposable income, marital status, and calendar year.

Among G1.5 immigrants, men had elevated mortality rates for all-site cancer (HR = 1.10; 95% CI, 1.06-1.14) and particularly for lung (HR = 1.32; 95% CI, 1.22-1.43), stomach (HR = 1.70; 95% CI, 1.45-1.99), and liver (HR = 1.34; 95% CI, 1.14-1.56) cancers compared with native Swedes. Conversely, mortality rates for melanoma (HR = 0.65; 95% CI, 0.52-0.83) and brain and CNS cancers (HR = 0.81; 95% CI, 0.69-0.95) were lower in this group. In female G1.5 immigrants, mortality rate was significantly higher only for stomach cancer (HR = 1.78; 95% CI, 1.50-2.12), whereas the rates were lower for all-site cancer (HR = 0.92; 95% CI, 0.88-0.95) and, in particular, for breast (HR = 0.86; 95% CI, 0.78-0.93), colorectal (HR = 0.88; 95% CI, 0.79-0.97), melanoma (HR = 0.53; 95% CI, 0.40-0.69), brain and CNS (HR = 0.71; 95% CI, 0.58-0.86), and ovarian (HR = 0.81; 95% CI, 0.71-0.94) cancers compared with the rate of native Swedes (Table 2).

For most cancer sites, mortality rates did not significantly differ among G2 immigrants compared with native Swedes; however, elevated mortality rates were observed in men for all-site cancers (HR = 1.04; 95% CI, 1.02-1.07) and cancer of the liver (HR = 1.30; 95% CI, 1.16-1.45) and lymphohematopoietic cancers (HR = 1.08; 95% CI, 1.00-1.18); and, in women, for cancers of the lung (HR = 1.09; 95% CI, 1.03-1.16), stomach (HR = 1.27; 95% CI, 1.08-1.51), and cervix uteri (HR = 1.18; 95% CI, 1.01-1.38) (Table 2).

### Region and country-specific estimates

**Figure 2:**
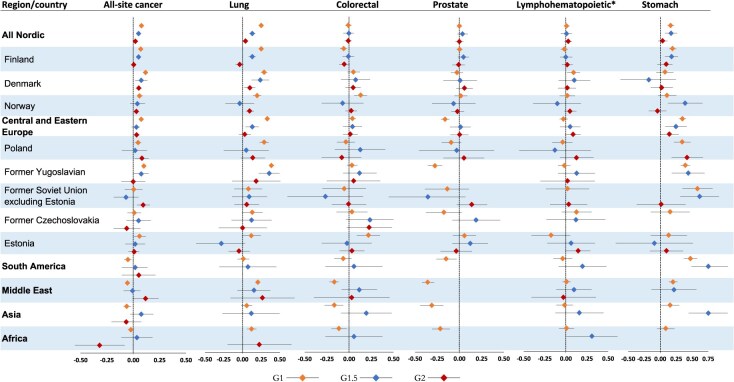
Logarithm of hazard ratios (HRs) with 95% CI bars for all-site and site-specific cancer deaths among male immigrant generations by country of origin, compared with natives, Sweden, 1990-2023. The exponentiated log HRs are available in Table S1. The HRs for cancer types not reported for regions or countries with fewer than 5 deaths for the respective cancers. *Includes leukemia, multiple myeloma, and Hodgkin and non-Hodgkin lymphoma. G1, adult (arrived when aged ≥18 years) first-generation immigrants; G1.5, child (arrived when aged <18 years) first-generation immigrants; G2, children of immigrants born in Sweden (second-generation immigrants).

**Figure 3:**
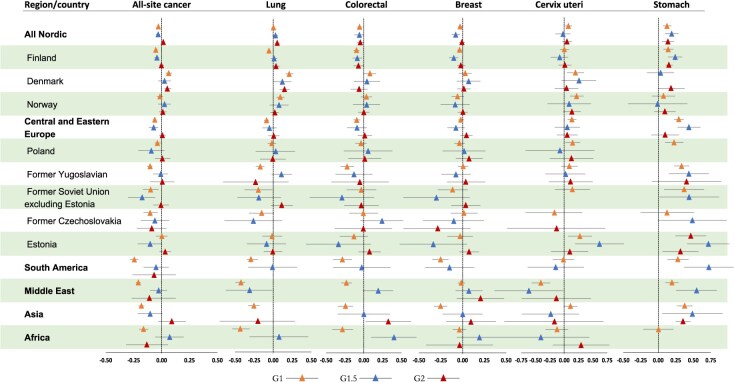
Logarithm of hazard ratios (HRs) with 95% CI bars for all-site and site-specific cancer deaths among female immigrant generations by country of origin, compared with natives, Sweden, 1990-2023. The exponentiated log HRs are available in Table S2. The HRs for cancer types not reported for regions or countries with fewer than 5 deaths for the respective cancers. G1, adult (arrived when aged ≥18 years) first-generation immigrants; G1.5, child (arrived when aged <18 years) first-generation immigrants; G2, children of immigrants born in Sweden (second-generation immigrants).

Figure 2 (men) and Figure 3 (women) present adjusted HRs with a forest plot for all-site cancer and selected cancer types by country of origin. The corresponding HRs with 95% CIs are presented in Tables S1 and S2.

### All-site cancer mortality

The all-site cancer mortality rate was elevated in G1 immigrant men from Nordic (HR = 1.20; 95% CI, 1.17-1.22) and other European countries, particularly central and eastern Europe countries (HR = 1.19; 95% CI, 1.15-1.22); conversely, it was lower in non-Western G1 immigrant men (HR = 0.89; 95% CI, 0.86-0.92) compared with native Swedish men. G1.5 immigrant men from Finland (HR = 1.12; 95% CI, 1.07-1.18), Denmark (HR = 1.19; 95% CI, 1.04-1.36), and former Yugoslavia (HR = 1.19; 95% CI, 1.01-1.40), as well as G2 immigrant men with Nordic (HR = 1.05; 95% CI, 1.02-1.8), Polish (HR = 1.21; 95% CI, 1.03-1.40), and former Soviet Union (HR = 1.24; 95% CI, 1.08-1.44) parental origins had elevated all-site cancer mortality rates compared with the rate of native Swedes (Figure 2; Table S1).

For most country-level estimates, the all-site cancer mortality rate was lower in G1 immigrant females compared to the rate of native Swedes. An exception was female Danish G1 immigrants who had a 16% increased risk of all-site cancer mortality (HR = 1.16; 95% CI, 1.09-1.24). Among female G1.5 immigrants, all-site cancer mortality rates were generally lower for most country-specific estimates compared with native Swedes; however, those from Africa had an 18% elevated rate of all-site cancer mortality, although this difference was not statistically significant. Only female G2 immigrants with Danish parental origin exhibited a significantly higher risk of all-site cancer mortality compared with native Swedish women (HR = 1.13; 95% CI, 1.06-1.22) (Figure 3; Table S2).

### Lung cancer

In all generation groups, the rate of lung cancer mortality was significantly higher for Nordic men (HR = 1.79 [95% CI, 1.71-1.88] in G1; HR = 1.35 [95% CI, 1.23-1.49] in G1.5; and HR = 1.10 [95% CI, 1.02-1.19] in G2) compared with native Swedish men. Lung cancer mortality also was elevated in male G1 and G1.5 immigrants from Europe (HR = 1.85 [95% CI, 1.76-1.93] and 1.32 [95% CI, 1.13-1.53], respectively), male G1 immigrants from the Middle East (HR = 1.61; 95% CI, 1.50-1.73) and Africa (HR = 1.33; 95% CI, 1.15-1.53), and male G2 immigrants with Asian parental origin (HR = 1.93; 95% CI, 1.00-3.70) compared with native Swedes (Figure 2; Table S1). Except for Nordic immigrants, lung cancer mortality rates were either significantly lower or were not notably different for most country-level estimates among female G1 and G1.5 immigrants compared with native Swedish women (Figure 3; Table S2).

### Colorectal cancer

The colorectal cancer mortality rate was elevated in male G1 immigrants from Norway (HR = 1.35; 95% CI, 1.14-1.59) and Estonia (HR = 1.65; 95% CI, 1.22-2.23), whereas the rate was lower among male G1 immigrants from non-Western countries compared with native Swedish men (Figure 2; Table S1). In women, colorectal cancer mortality rates were elevated among Danish G1 immigrants (HR = 1.21; 95% CI, 1.01-1.45), G1 immigrants from Africa (HR = 2.51; 95% CI, 1.25-5.03), and G2 immigrants with Asian parental origin (HR = 2.13; 95% CI, 1.06-4.26); conversely, Finish immigrants consistently had a 10% to 15% lower mortality rate of colorectal cancer across the generation groups (Figure 3; Table S2).

### Breast and cervix uteri cancers

Breast cancer mortality rate was significantly lower in Finnish G1 (HR = 0.93; 95% CI, 0.87-0.99) and G1.5 (HR = 0.79; 95% CI, 0.70-0.89) immigrants, as well as in non-Western G1 immigrants (HR = 0.81; 95% CI, 0.75-0.87). However, G2 immigrants with European parental origin had an elevated mortality rate for breast cancer compared with native Swedish women (HR = 1.21; 95% CI, 1.09-1.34). Cervix uteri cancer mortality was higher among G1 immigrants from Nordic countries (HR = 1.19; 95% CI, 1.03-1.38), Poland (HR = 1.42; 95% CI, 1.04-1.90), and Estonia (HR = 1.88; 95% CI, 1.37-3.04), as well as some immigrant offspring (G2) with Nordic and European parental origin, compared with native Swedes, although these estimates did not reach statistical significance (Figure 3; Table S2).

### Prostate cancer

The rate of prostate cancer mortality did not significantly differ in G1.5 and G2 compared with native Swedes. However, lower mortality rates were observed in G1 immigrants from Europe (HR = 0.72; 95% CI, 0.66-0.78) and from non-Western countries (HR = 0.51; 95% CI, 0.46-0.57) (Figure 2; Table S1).

### Lymphohematopoietic cancers

Danish G1, non-Western G1.5, and G2 individuals with European parental origin had elevated mortality rates of lymphohematopoietic cancers (HR = 1.24 [95% CI, 1.03-1.43]; HR = 1.46 [95% CI, 1.07-1.98]; and HR = 1.21 [95% CI, 1.05-1.39], respectively) compared with native Swedish men. Apart from these groups, there were no significant differences in lymphohematopoietic cancer mortality rates across male generation groups at the country and region levels (Figure 2; Table S1).

### Stomach cancer

G1 and G1.5 immigrants had elevated mortality rates of stomach cancer in most country-specific estimates. In G2 immigrants, men from Poland (HR = 2.64; 95% CI, 1.50-4.67) and women from Nordic countries (HR = 1.38; 95% CI, 1.13-1.62), Estonia (HR = 2.08; 95% CI, 1.15-3.76), and non-Western countries (HR = 2.53; 95% CI, 1.12-6.68), parental origin was associated with higher stomach cancer mortality rates compared with those of native Swedes.

## Discussion

The present study provides evidence for disparities in overall and specific cancer mortality by country of birth among immigrant generations compared with that of native Swedes. Overall, the study revealed remarkable variations in cancer mortality by cancer type and country of origin across immigrant generations. In some cancers, high mortality rates were maintained across the immigrant generations, whereas for others, mortality rates varied substantially in G2 from the rate in G1 and G1.5, suggesting the transition in mortality rates across immigrant generations, which can be explained broadly by variations in biology (genetics) and other environmental and lifestyle risk factors, as well as factors determining postdiagnosis survival rates, such as differences in access and quality of care and treatment adherence.

The mortality advantage of colorectal, pancreatic (in females), and prostate cancers in G1 was not maintained in subsequent generations compared with the native population, a finding that can be attributed to various factors. No or little difference in postdiagnosis survival rates of these cancers between immigrants and nonimmigrants was reported in previous studies.^25^ However, differences in cancer risk profile among immigrant generations, such as changes in health behaviors, could remarkably contribute to the observed disparities. Health behaviors, including physical activity, dietary intake, alcohol consumption, and tobacco smoking, are crucial, given their substantial impact on cancer risk and death, accounting for up to 50% of cases. Although lifestyle changes are influenced by the context and composition of the immigrant population in the receiving countries,^26^ immigrants tend to change their lifestyle in the host country^26–28^ and may pass these changes to the subsequent generations,^29^ suggesting the convergency of behavioral risk factors toward the native population.

In the present study, we also noted that the lung cancer mortality rate in women was lower in G1, but increased in G2, compared with native Swedish women. In contrast, the higher mortality rate of lung cancer among G1 men decreased in G2 compared with the rate in native Swedish men. Differences in the stage at diagnosis and pattern of cancer care between immigrants and nonimmigrants in Sweden are minor,^30^ so their contributions to lung cancer mortality disparities is likely minimal; instead, disparities may be strongly linked to variations in risk factors. Cigarette smoking is the primary risk factor for the incidence and mortality of lung cancer.^31^ Although Sweden is among the countries with a decreasing pattern in the prevalence of cigarette smoking rate (~ 6% daily smokers in 2021, decreased by half from the rate in 2012), the proportion of smokers is significantly high among foreign-born men and women, especially immigrants from Nordic and other European countries, compared with native Swedes.^32^^,^^33^ Thus, the high smoking rate among immigrants, augmented by the likelihood of children of immigrants inheriting their parents’ smoking habit, as well as being a passive smoker at home, may explain the observed heightened risk of lung cancer mortality in both G1 and G2 immigrants compared with native Swedes.

Another main finding of the study was that, despite some exceptions, the disparities in cancer mortality rates between G2 and native Swedes were less remarkable, particularly for cancers with lower mortality rates in G1. This suggests that the presence of the healthy migrant effect diminishes in subsequence generations. The findings are aligned with previous studies that showed a resemblance in mortality rates between natives and G2 immigrants.^10^^,^^12^^,^^15^^,^^34^ This phenomenon may be attributed to several factors related to acculturation and cultural assimilation to the host’s country environment, as well as improved access to cancer screening and health care among individuals born and raised in the host country. However, for some cancers, like cervix uteri cancer, the risk of mortality remained elevated in G2 immigrants, particularly among those whose parent(s) were born in the Nordic region. A previous study in Sweden showed that Nordic female immigrants, except those who are Finnish, have a higher risk of cervix uteri cancer and a lower attendance rate for the national recommendation of cervical screening, compared with Swedish-born women.^35^ Mousavi et al.^36^ also revealed that the higher risk of gynecological cancers, including cervix uteri cancer, in G1 immigrants, particularly in Danish, Norwegian, and East European immigrants in Sweden, was also evident in G2 immigrants. Thus, the elevated mortality rate of cervix uteri cancer observed among G2 female immigrants with Nordic backgrounds in our study may be linked to their lower participation in cervical screening, contributing to higher incidence and diagnosis at later stage of cervix uteri cancer in this group.

This study highlighted that the elevated mortality rate of liver cancer (in men) and stomach cancer (in women) among G1, compared with native Swedes, remained in the subsequent generations (G1.5 and G2). In line with our findings, elevated mortality rates of liver and stomach cancers were reported among G1 immigrants in many studies, both in Sweden^37^^,^^38^ and other countries,^15^^,^^39–41^ compared with the host population. These mortality disparities likely may be explained by the risk factors; the studies’ authors suggested postdiagnosis survival rates of liver and stomach cancers were similar or even better among immigrants than in the host population.^25^ The strongest risk factors for liver and stomach cancers are, respectively, infections with hepatitis B virus (HBV), hepatitis C virus,^42^ and *Helicobacter pylori*.^43^ These infections are typically contracted during childhood in endemic regions, particularly in developing countries where most recent immigrants originated. Consequently, premigration factors such as delayed detection and treatment of these infections, mostly due to limited access to health care, may account for the disparities observed in G1 and G1.5 immigrants, especially those from non-Western regions and eastern and southern European countries, where infection rates are high. Acquisition of *H. pylori* infection can be possible within a family, although the main mode of transmission remains uncertain. Studies suggest transmission mostly occurs from mother to child at early childhood age. Konno et al.^44^ reported that the rate of *H. pylori* acquisition among children born to *H. pylori*–positive mothers was 11% from birth to 5 years of age. Likewise, HBV can be transmitted from mothers with chronic HBV infection to their babies during delivery (so-called vertical transmission),^45^ which has a higher risk of subsequent liver diseases and mortality compared with HBV acquisition later in life via a horizontal transmission mode, such as drug injections and heterosexual contact. Most people with chronic HBV infection in Sweden are non-Western immigrants who likely were infected early in life or before migrating to Sweden.^46^ Therefore, the elevated mortality rates of liver and stomach cancer in G2 immigrants could potentially be attributed to the possibility of infections acquired within a family, but also modulated by other behavioral factors. For example, eating processed and grilled meat, smoking, excessive alcohol, and other environmental factors could explain the elevated mortality rate of stomach cancer (possibly for noncardiac type) in the Nordic region, because the prevalence of *H. pylori* infections in this region is low. The World Health Organization adopted the Global Health Sector Strategy in 2016 to eliminate viral hepatitis as a major public health threat by 2030.^47^ In Sweden, both HBV and hepatitis C virus infections are notifiable by law, with a national screening program for pregnant women and vaccination for all children and those born to HBV-positive mothers at birth.^46^ Nevertheless, some studies questioned the sufficiency of HBV vaccination to prevent mother-to-child transmission.^48^ Although the notification rate of HBV infection decreased by 40% between 2015 and 2018 in Sweden,^49^ continued immigration requires strengthened HBV surveillance for immigrants from HBV-endemic regions upon their arrival.

We also noted that despite sociodemographic characteristics, including education and income, are strong contributing factors for cancer mortality disparities between immigrants and nonimmigrants,^18^ in our study, adjustment for these characteristics modified the association to a significant degree only in women, suggesting that socioeconomic disparities tend to be less pronounced in male immigrant generations than those in female immigrants. This might be because female immigrants face multiple layers of disadvantage, including lower employment and wage rates, as well as greater cultural barriers than their male counterparts. Because cancer mortality disparities are likely influenced by a complex interplay of multiple factors beyond the sociodemographic characteristics, considering other confounding factors is essential, especially when assessing disparities among male immigrants.

Overall, our study provides comprehensive evidence of disparities in overall and specific cancer mortality by immigrant generations and country of origin. These disparities can be reflected mainly in their incidence rates, particularly for high-fatality cancers, such as pancreatic, liver, lung, and ovarian cancers, which are often diagnosed at advanced stages with limited treatment options. Interpretation of the findings need to consider this, as well as the following limitations. First, we did not account for emigrants who leave the country near the time of death (commonly known as the “salmon bias”), particularly in G1 immigrants, and this may underestimate mortality rates among immigrants. However, because some studies have suggested that higher disease severity was associated with less emigration, it is unlikely that the salmon bias affects our conclusion.^50^ This could also hold for cancer. Unlike the comprehensive insurance scheme available in Sweden, cancer care including expensive diagnostic and treatment services is not freely available in most immigrant countries of origin. Second, although we used a longer period with an older sample of G1.5 and G2 immigrants, these groups are relatively young and their total numbers are small. This affects the number of observed cases (ie, cancer deaths), leading to low statistical power in some estimates (ie, they may not be robust), especially when these groups are further disaggregated by country of origin. Third, in this study, we did not account for other important factors that could further modify mortality disparities across immigrant generations, such as lifestyle and medical-related data.

In conclusion, cancer mortality rates vary by immigrant generation and their country of origin, despite disparities being less pronounced among G2 immigrants than among native Swedes. Transitions of mortality risk across the immigrant generations were noted for most cancers; however, the elevated risk of mortality for cancers linked to infections such as liver and stomach cancers remained in all immigrant generations; thus, surveillance of infectious diseases related to cancer among immigrant populations in Sweden should be enhanced. Generational status could modify cancer mortality risks among immigrants and can be used to guide target groups for cancer prevention and control interventions.

## Supplementary material

Supplementary material is available at the *American Journal of Epidemiology* online.

## Supplementary Material

Web_Material_kwae388

## Data Availability

Ethical approval from the Swedish ethical review agency is necessary to address the research question, and data can be requested from the responsible authority. Because of the sensitive nature of individual-level health data, restrictions are in place. However, data may be obtainable from the authors upon reasonable request and with permission from the responsible authority.
