# Supplementary material for: Disparities in overall and site-specific cancer mortality among immigrant generations in Sweden: a nationwide follow-up study over 3 decades
Source: Am J Epidemiol. 2024 Oct 4;194(8):2325–35. doi: 10.1093/aje/kwae388 (PMC12342873; doi:10.1093/aje/kwae388)
Supplement: Web_Material_kwae388 [file web_material_kwae388.docx]

**Supplemental material**

**Disparities in overall and site-specific cancer mortality among immigrant generations in Sweden: A nationwide follow-up study over three decades**

Daniel Nigusse Tollosa, Kazem Zendehdel , Paolo Boffetta^,^, Eero Pukkala, Mikael Rostila

**Table of contents**

**Table S1:** Cancer mortality among males by country of origin and immigrant generations for overall and selected cancer types in Sweden, 1990-2023. Corresponding HRs are plotted in figure 2

**Table S2:** Cancer mortality among females by country of origin and immigrant generations for overall and selected cancer types in Sweden, 1990-2023. Corresponding HRs are plotted in figure 3

**Table S1 -** Cancer mortality among males by country of origin and immigrant generations for overall and selected cancer types in Sweden, 1990-2023. Corresponding HRs are plotted in figure 2

| **Region/Country** | **Generation** | **Person years** | **All-sites** | | **Lung** | | **Colorectal** | | **Prostate** | | **Lymphohematopoietic** | | **Stomach** | |
| --- | --- | --- | --- | --- | --- | --- | --- | --- | --- | --- | --- | --- | --- | --- |
|  |  |  | Cases | HR (95% CI) | Cases | HR (95% CI) | Cases | HR (95% CI) | Cases | HR (95% CI) | Cases | HR (95% CI) | Cases | HR (95% CI) |
| **NATIVES (Ref)** | - | 72,175,976 | 115, 654 | 1.00 | 18,999 | 1.00 | 14,376 | 1.00 | 15,180 | 1.00 | 10,956 | 1.00 | 3,769 | 1.00 |
| **ALL NORDIC** | G1 | 2,582,793 | 14,016 | 1.20 (1.17, 1.22) | 3,609 | 1.79 (1.71, 1.88) | 1,413 | 0.98 (0.91, 1.06) | 2,160 | 1.00 (0.94, 1.07) | 1,097 | 1.02 (0.94, 1.11) | 588 | 1.46 (1.30, 1.65) |
|  | G1.5 | 1,213,027 | 2,051 | 1.12 (1.08, 1.18) | 408 | 1.35 (1.23, 1.49) | 222 | 0.99 (0.86, 1.13) | 231 | 1.08 (0.95, 1.24) | 168 | 1.02 (0.87, 1.18) | 91 | 1.48 (1.19, 1.83) |
|  | G2 | 4,720,078 | 4,088 | 1.05 (1.02, 1.08) | 641 | 1.10 (1.02, 1.19) | 489 | 0.98 (0.89, 1.07) | 374 | 1.00 (0.90, 1.11) | 395 | 1.07 (0.96, 1.18) | 141 | 1.09 (0.92, 1.29) |
| Finland | G1 | 1,519,656 | 8,173 | 1.18 (1.15, 1.21) | 2,214 | 1.79 (1.70, 1.89) | 727 | 0.87 (0.79, 0.95) | 1,186 | 1.01 (0.93, 1.09) | 624 | 0.96 (0.87, 1.06) | 386 | 1.56 (1.37, 1.79) |
|  | G1.5 | 932,649 | 1,675 | 1.12 (1.07, 1.18) | 335 | 1.35 (1.21, 1.50) | 181 | 0.98 (0.84, 1.14) | 194 | 1.11 (0.96, 1.28) | 137 | 1.00 (0.85, 1.19) | 76 | 1.50 (1.19, 1.89) |
|  | G2 | 3,087,983 | 1,909 | 1.01 (0.95, 1.06) | 244 | 0.92 (0.81, 1.04) | 216 | 0.88 (0.77, 1.01) | 154 | 0.98 (0.83, 1.15) | 193 | 1.04 (0.90, 1.21) | 78 | 1.24 (0.9, 1.55) |
| Denmark | G1 | 540,263 | 3,279 | 1.31 (1.23, 1.37) | 844 | 1.95 (1.76, 2.16) | 349 | 1.12 (0.96, 1.32) | 499 | 0.94 (0.80, 1.10) | 264 | 1.24 (1.03, 1.47) | 93 | 1.19 (0.87, 1.61) |
|  | G1.5 | 150,960 | 222 | 1.19 (1.04, 1.36) | 52 | 1.73 (1.32, 2.27) | 27 | 1.18 80.81, 1.72) | 21 | 1.02 (0.66, 1.58) | 21 | 1.26 (0.82, 1.94) | 4 | 0.66 (0.24, 1.75) |
|  | G2 | 709,937 | 778 | 1.13 (1.05, 1.21) | 131 | 1.26 (1.06, 1.49) | 98 | 1.11 (0.91, 1.35) | 79 | 1.14 (0.92, 1.42) | 68 | 1.04 (0.81, 1.32) | 24 | 1.05 (0.71, 1.58) |
| Norway | G1 | 471,440 | 2,487 | 1.15 (1.08, 1.23) | 528 | 1.57 (1.38, 1.78) | 330 | 1.35 (1.14, 1.59) | 467 | 1.04 (0.88, 1.23) | 203 | 1.04 (0.84, 1.28) | 106 | 1.28 (0.93, 1.78) |
|  | G1.5 | 112,483 | 150 | 1.09 (0.93, 1.29) | 21 | 0.92 (0.59, 1.44) | 14 | 0.84 (0.49, 1.45) | 16 | 0.86 (0.49, 1.52) | 9 | 0.79 (0.41, 1.52) | 11 | 2.45 (1.32, 4.57) |
|  | G2 | 903,257 | 1,399 | 1.07 (1.02, 1.13) | 266 | 1.25 (1.11, 1.42) | 175 | 1.06 (0.91, 1.23) | 141 | 0.95 (0.81, 1.13) | 134 | 1.12 (0.95, 1.33) | 39 | 0.91 (0.66, 1.25) |
| **All EUROPE** | G1 | 4,127,589 | 12,678 | 1.11 (1.08, 1.13) | 3,110 | 1.85 (1.76, 1.93) | 1,422 | 1.01 (0.94, 1.09) | 1,710 | 0.72 (0.66, 0.78) | 1,054 | 0.95 (0.87, 1.03) | 761 | 2.03 (1.84, 2.24) |
|  | G1.5 | 1,004,231 | 938 | 1.06 (0.98, 1.13) | 182 | 1.32 (1.13, 1.53) | 117 | 1.08 (0.90, 1.31) | 94 | 0.83 (0.67, 1.04) | 93 | 1.14 (0.93, 1.40) | 52 | 1.67 (1.25, 2.25) |
|  | G2 | 2,621,076 | 1,767 | 1.03 (0.98, 1.08) | 253 | 1.05 (0.95, 1.19) | 219 | 0.98 (0.86, 1.12) | 157 | 0.97 (0.83, 1.14) | 202 | 1.21 (1.05, 1.39) | 65 | 1.17 (0.91, 1.49) |
| **Central and Eastern Europe** | G1 | 2,188,466 | 7,289 | 1.19 (1.15, 1.22) | 1,902 | 2.15 (2.03, 2.27) | 826 | 1.09 (1.01, 1.19) | 891 | 0.69 (0.62, 0.77) | 576 | 0.94 (0.84, 1.05) | 470 | 2.23 (1.96, 2.52) |
|  | G1.5 | 668,885 | 522 | 1.07 (0.96, 1.16) | 104 | 1.35 (1.10, 1.65) | 62 | 1.09 (0.85, 1.40) | 61 | 1.03 (0.78, 1.34) | 51 | 1.12 (0.85, 1.48) | 32 | 1.76 (1.20, 2.59) |
|  | G2 | 1,229,607 | 816 | 1.08 (1.01, 1.15) | 115 | 1.08 (0.90, 1.29) | 101 | 1.03 (0.85, 1.25) | 68 | 1.01 (0.79, 1.27) | 88 | 1.22 (0.98, 1.50) | 34 | 1.39 (0.99, 1.95) |
| Poland | G1 | 611,834 | 1,230 | 1.11 (1.03, 1.19) | 286 | 1.95 (1.69, 2.25) | 141 | 0.92 (0.73, 1.16) | 145 | 0.81 (0.63, 1.05) | 116 | 0.92 (0.69, 1.20) | 78 | 2.21 (1.64, 2.98) |
|  | G1.5 | 149,231 | 63 | 1.04 (0.78, 1.35) | 13 | 1.12 (0.56, 2.24) | 9 | 1.33 (0.69, 2.56) | 4 | 0.93 (0.35, 2.47) | 5 | 0.74 (0.28, 1.98) | 2 | - |
|  | G2 | 252,350 | 169 | 1.21 (1.03, 1.40) | 28 | 1.38 (0.95, 2.01) | 15 | 0.83 (0.49, 1.37) | 14 | 1.13 (0.66, 1.91) | 18 | 1.34 (0.85, 2.13) | 12 | 2.64 (1.50, 4.67) |
| Former Yugoslavian | G1 | 742,468 | 2,537 | 1.26 (1.21, 1.31) | 855 | 2.45 (2.27, 2.65) | 256 | 1.08 (0.95, 1.24) | 172 | 0.53 (0.44, 0.64) | 182 | 0.96 (0.81, 1.13) | 172 | 2.47 (2.08, 2.94) |
|  | G1.5 | 294,177 | 142 | 1.19 (1.01, 1.40) | 38 | 2.27 (1.65, 3.13) | 20 | 1.31 (0.85, 2.04) | 3 | - | 15 | 1.34 (0.81, 2.22) | 12 | 2.73 (1.51, 4.95) |
|  | G2 | 366,295 | 59 | 1.00 (0.77, 1.30) | 7 | 1.53 (0.73, 3.23) | 8 | 1.13 (0.56, 2.25) | 1 | - | 8 | 1.05 (0.50, 2.21) | 2 | - |
| Former Soviet Union | G1 | 61,279 | 347 | 1.01 (0.83, 1.22) | 55 | 1.20 (0.78, 1.84) | 42 | 0.88 (0.50, 1.55) | 64 | 0.73 (0.41, 1.28) | 31 | 1.05 (0.58, 1.89) | 30 | 3.83 (2.22, 6.61) |
|  | G1.5 | 30,466 | 60 | 0.85 (0.65, 1.12) | 15 | 1.23 (0.72, 2.13) | 4 | 0.54 (0.20, 1.44) | 5 | 0.44 (0.16, 1.17) | 2 | - | 9 | 4.13 (2.06, 8.28) |
|  | G2 | 89,486 | 186 | 1.24 (1.08, 1.44) | 27 | 1.14 (0.78, 1.67) | 19 | 0.99 (0.64, 1.56) | 25 | 1.38 (0.93, 2.05) | 15 | 1.08 (0.65, 1.79) | 5 | 1.04 (0.43, 2.49) |
| Former Czechoslovakia | G1 | 72,620 | 425 | 1.02 (0.88, 1.18) | 76 | 1.36 (0.99, 1.86) | 59 | 1.08 (0.72, 1.61) | 63 | 0.67 (0.42, 1.06) | 48 | 1.34 (0.89, 2.02) | 24 | 1.43 (0.71, 2.88) |
|  | G1.5 | 30,059 | 59 | 1.12 (0.86, 1.47) | 10 | 1.32 (0.71, 2.46) | 10 | 1.71 (0.92, 3.17) | 12 | 1.54 (0.83, 2.88) | 7 | 1.32 (0.59, 2.95) | 3 | - |
|  | G2 | 79,658 | 43 | 0.87 (0.65, 1.17) | 7 | 1.01 (0.48, 2.11) | 11 | 1.69 (0.94, 3.06) | 3 | - | 2 | - | 2 | - |
| Estonia | G1 | 118,668 | 1,103 | 1.15 (1.02, 1.31) | 183 | 1.32 (0.99, 1.75) | 146 | 1.65 (1.22, 2.23) | 251 | 1.14 (0.84, 1.54) | 72 | 0.67 (0.39, 1.12) | 73 | 1.36 (0.71, 2.62) |
|  | G1.5 | 23,015 | 97 | 1.04 (0.84, 1.29) | 9 | 0.52 (0.24, 1.09) | 9 | 0.94 (0.49, 1.80) | 24 | 1.32 (0.83, 2.10) | 9 | 1.15 (0.60, 2.21) | 4 | 0.81 (0.20, 3.25) |
|  | G2 | 223,007 | 270 | 1.02 (0.90, 1.14) | 36 | 0.90 (0.65, 1.25) | 38 | 1.08 (0.78, 1.49) | 23 | 0.92 (0.61, 1.38) | 34 | 1.40 (1.00, 1.96) | 11 | 1.26 (0.69, 2.27) |
| **Rest of Europe** | G1 | 1,939,123 | 5,389 | 1.01 (0.97, 1.05) | 1,208 | 1.48 (1.28, 1.59) | 596 | 0.92 (0.83, 1.03) | 819 | 0.75 (0.67, 0.84) | 478 | 0.96 (0.86, 1.08) | 291 | 1.78 (1.53, 2.07) |
|  | G1.5 | 335,345 | 416 | 1.05 (0.95, 1.16) | 78 | 1.27 (1.02, 1.59) | 55 | 1.08 (0.81, 1.42) | 33 | 0.63 (0.43, 0.91) | 42 | 1.16 (0.85, 1.58) | 20 | 1.57 (1.00, 2.47) |
|  | G2 | 1,391,468 | 951 | 0.98 (0.92, 1.05) | 138 | 1.03 (0.87, 1.22) | 118 | 0.94 (0.78, 1.13) | 89 | 0.96 (0.77, 1.17) | 114 | 1.20 (0.99, 1.45) | 31 | 0.99 (0.70, 1.42) |
| Germany | G1 | 491,709 | 2,294 | 1.04 (0.98, 1.10) | 434 | 1.23 (1.08, 1.41) | 295 | 1.18 (1.01, 1.37) | 404 | 0.85 (0.72, 0.99) | 194 | 0.92 (0.75, 1.11) | 104 | 1.37 (1.03, 1.81) |
|  | G1.5 | 112,280 | 235 | 1.07 (0.94, 1.23) | 37 | 1.07 (0.77, 1.48) | 33 | 1.16 (0.81, 1.67) | 15 | 0.47 (0.27, 0.81) | 23 | 1.21 (0.80, 1.81) | 5 | 0.77 (0.32, 1.84) |
|  | G2 | 726,069 | 610 | 0.98 (0.90, 1.06) | 98 | 1.10 (0.91, 1.35) | 81 | 0.99 (0.80, 1.23) | 59 | 0.93 (0.72, 1.20) | 68 | 1.12 (0.87, 1.42) | 20 | 0.99 (0.64, 1.53) |
| **NON-WESTERN COUNTRIES** | G1 | 6,562,803 | 6,114 | 0.89 (0.86, 0.92) | 1,493 | 1.42 (1.34, 1.51) | 599 | 0.71 (0.65, 0.77) | 481 | 0.51 (0.46, 0.57) | 628 | 0.99 (0.91, 1.09) | 394 | 1.68 (1.49, 1.89) |
|  | G1.5 | 1,855,192 | 256 | 1.05 (0.92, 1.18) | 25 | 1.19 (0.80, 1.77) | 39 | 1.30 (0.94, 1.78) | 3 | - | 45 | 1.46 (1.07, 1.98) | 26 | 3.47 (2.33, 5.16) |
|  | G2 | 1,059,050 | 129 | 0.96 (0.80, 1.14) | 18 | 1.52 (0.95, 2.41) | 10 | 0.66 (0.35, 1.23) | 6 | 0.77 (0.34, 1.71) | 16 | 0.90 (0.54, 1.51) | 4 | 1.12 (0.42, 3.00) |
| **South America** | G1 | 535,631 | 782 | 0.89 (0.82, 0.96) | 149 | 1.03 (0.86, 1.23) | 84 | 0.86 (0.68, 1.07) | 90 | 0.71 (0.55, 0.93) | 71 | 0.92 (0.71, 1.19) | 84 | 2.97 (2.33, 3.78) |
|  | G1.5 | 304,545 | 50 | 1.04 (0.78, 1.36) | 5 | 1.18 (0.49, 2.85) | 7 | 1.13 (0.54, 2.37) | 0 | - | 9 | 1.57 (0.82, 3.03) | 9 | 5.92 (3.06, 11.45) |
|  | G2 | 172,736 | 30 | 1.13 (0.78, 1.62) | 1 | - | 2 | - | 2 | - | 2 | - | 2 | - |
| **Middle East** | G1 | 3,498,705 | 3,436 | 0.88 (0.84, 0.91) | 958 | 1.61 (1.50, 1.73) | 323 | 0.68 (0.60, 0.76) | 245 | 0.44 (0.38, 0.52) | 372 | 1.02 (0.91, 1.14) | 219 | 1.59 (1.36, 1.86) |
|  | G1.5 | 838,815 | 114 | 0.98 (0.81, 1.17) | 15 | 1.42 (0.86, 2.37) | 18 | 1.30 (0.82, 2.07) | 3 | - | 19 | 1.25 (0.77, 2.02) | 6 | 1.64 (0.73, 3.67) |
|  | G2 | 476,916 | 47 | 1.31 (0.98, 1.74) | 4 | 1.86 (0.69, 4.97) | 4 | 1.07 (0.40, 2.86) | 0 | - | 5 | 0.94 (0.39, 2.27) | 2 | - |
| **Asia** | G1 | 1,201,537 | 868 | 0.87 (0.81, 0.95) | 174 | 1.14 (0.96, 1.35) | 88 | 0.68 (0.53, 0.86) | 68 | 0.49 (0.36, 0.66) | 85 | 0.97 (0.77, 1.22) | 49 | 1.43 (1.03, 1.98) |
|  | G1.5 | 428,304 | 59 | 1.19 (0.93, 1.55) | 5 | 1.31 (0.54, 3.15) | 10 | 1.56 (0.81, 3.01) | 0 | - | 9 | 1.44 (0.75, 2.79) | 8 | 5.61 (2.78, 11.29) |
|  | G2 | 202,682 | 37 | 0.86 (0.62, 1.19) | 9 | 1.93 (1.00, 3.70) | 2 | - | 4 | 1.11 (0.42, 2.96) | 6 | 1.26 (0.56, 2.80) | 0 | - |
| **Africa** | G1 | 1,326,930 | 1,028 | 0.95 (0.89, 1.02) | 212 | 1.33 (1.15, 1.53) | 104 | 0.77 (0.63, 0.94) | 78 | 0.61 (0.49, 0.78) | 100 | 1.02 (0.83, 1.25) | 42 | 1.22 (0.89, 1.67) |
|  | G1.5 | 283,527 | 33 | 1.08 (0.77, 1.52) | 0 | - | 4 | 1.13 (0.54, 2.37) | 0 | - | 8 | 2.04 (1.02, 4.09) | 3 | - |
|  | G2 | 206,715 | 15 | 0.48 (0.28, 0.83) | 4 | 1.69 (0.63, 4.51) | 2 | - | 0 | - | 3 | - | 0 | - |
| **REST OF THE WORLD** | G1 | 211,983 | 201 | 0.94 (0.79, 1.11) | 40 | 1.30 (0.89, 1.90) | 12 | 0.54 (0.29, 1.01) | 35 | 1.19 (0.75, 1.89) | 23 | 1.08 (0.65, 1.79) | 9 | 0.88 (0.33, 2.34) |
|  | G1.5 | 50,527 | 19 | 1.31 (0.79, 2.16) | 2 | - | 3 | - | 3 | - | 3 | - | 3 | - |
|  | G2 | 91,686 | 261 | 1.11 (0.98, 1.25) | 41 | 0.99 (0.73, 1.35) | 30 | 1.09 (0.76, 1.56) | 61 | 1.48 (1.15, 1.91) | 22 | 0.91 (0.59, 1.39) | 7 | 0.94 (0.45, 1.97) |

HRs adjusted for age, educational status, disposable income, marital status, and calendar year – all characteristics measured at exit date of the study

**Table S2 -** Cancer mortality among females by country of origin and immigrant generations for overall and selected cancer types in Sweden, 1990 to 2023. Corresponding HRs are plotted in figure 3

| **Region/Country** | **Generation** | **Person years** | **All cancer** | | **Lung** | | **Colorectal** | | **Breast** | | **Stomach** | | **Cervical** | |
| --- | --- | --- | --- | --- | --- | --- | --- | --- | --- | --- | --- | --- | --- | --- |
|  |  |  | Cases | HR (95% CI) | Cases | HR (95% CI) | Cases | HR (95% CI) | Cases | HR (95% CI) | Cases | HR (95% CI) | Cases | HR (95% CI) |
| **NATIVES (Ref)** | - | 69,622,232 | 108,174 | 1.00 | 20,225 | 1.00 | 11,622 | 1.00 | 17,174 | 1.00 | 2167 | 1 | 1,797 | 1 |
| **ALL NORDIC** | G1 | 3,607,265 | 15,870 | 0.94 (0.92, 0.96) | 1763 | 1.01 (0.96, 1.06) | 825 | 0.88 (0.82, 0.95) | 1194 | 0.94 (0.89, 1.00) | 237 | 1.34 (1.17, 1.54) | 243 | 1.19 (1.03, 1.38) |
|  | G1.5 | 1,376,899 | 2,440 | 0.93 (0.89, 0.97) | 528 | 1.07 (0.98, 1.16) | 229 | 0.87 (0.75, 0.99) | 336 | 0.83 (0.75, 0.93) | 79 | 1.56 (1.24, 1.95) | 45 | 0.95 (0.70, 1.27) |
|  | G2 | 4,495,839 | 4047 | 1.04 (1.00, 1.07) | 790 | 1.13 (1.06, 1.22) | 363 | 0.90 (0.81, 1.01) | 700 | 0.99 (0.92, 1.07) | 105 | 1.38 (1.13, 1.68) | 107 | 1.12 (0.92, 1.36) |
| Finland | G1 | 2,444,514 | 10,155 | 0.89 (0.87, 0.92) | 1171 | 0.88 (0.83, 0.93) | 561 | 0.80 (0.73, 0.87) | 888 | 0.93 (0.87, 0.99) | 188 | 1.39 (1.19, 1.63) | 142 | 1.01 (0.84, 1.21) |
|  | G1.5 | 1,080,681 | 1,950 | 0.91 (0.86, 0.95) | 423 | 1.03 (0.93, 1.13) | 180 | 0.82 (0.71, 0.95) | 267 | 0.79 (0.70, 0.89) | 73 | 1.75 (1.38, 2.21) | 32 | 0.83 (0.58, 1.17) |
|  | G2 | 2,948,513 | 1966 | 1.00 (0.95, 1.05) | 362 | 1.09 (0.98, 1.21) | 167 | 0.85 (0.73, 0.99) | 360 | 0.96 (0.87, 1.07) | 54 | 1.43 (1.08, 1.86) | 58 | 1.03 (0.79, 1.34) |
| Denmark | G1 | 459,851 | 2,450 | 1.16 (1.09, 1.24) | 288 | 1.63 (1.45, 1.84) | 121 | 1.21 (1.01, 1.45) | 145 | 1.08 (0.91, 1.27) | 21 | 1.08 (0.69, 1.68) | 38 | 1.57 (1.12, 2.21) |
|  | G1.5 | 145,868 | 239 | 1.06 (0.93, 1.21) | 55 | 1.32 (1.01, 1.72) | 24 | 1.10 (0.74, 1.64) | 40 | 1.17 (0.86, 1.60) | 2 | - | 8 | 1.81 (0.90, 3.63) |
|  | G2 | 671,827 | 764 | 1.13 (1.06, 1.22) | 173 | 1.41 (1.21, 1.64) | 60 | 0.87 (0.67, 1.12) | 125 | 1.04 (0.87, 1.24) | 20 | 1.53 (0.99, 2.38) | 17 | 1.10 (0.68, 1.77) |
| Norway | G1 | 652,680 | 3,176 | 0.97 (0.91, 1.03) | 280 | 1.24 (1.10, 1.40) | 134 | 1.09 (0.91, 1.29) | 150 | 0.88 (0.75, 1.04) | 28 | 1.19 (0.81, 1.74) | 59 | 1.66 (1.26, 2.19) |
|  | G1.5 | 132,368 | 245 | 1.06 (0.93, 1.21) | 48 | 1.19 (0.90, 1.59) | 24 | 1.09 (0.72, 1.64) | 27 | 0.82 (0.56, 1.21) | 4 | 0.98 (0.37, 2.61) | 5 | 1.21 (0.51, 2.93) |
|  | G2 | 858,273 | 1309 | 1.03 (0.95, 1.11) | 254 | 1.05 (0.92, 1.18) | 135 | 1.00 (0.84, 1.19) | 213 | 1.01 (0.88, 1.15) | 31 | 1.25 (0.88, 1.78) | 32 | 1.37 (0.96, 1.95) |
| **ALL EUROPE** | G1 | 4,586,094 | 11,156 | 0.84 (0.82, 0.86) | 1062 | 0.77 (0.72, 0.82) | 640 | 0.83 (0.76, 0.90) | 1053 | 0.95 (0.89, 1.01) | 267 | 1.86 (1.63, 2.12) | 203 | 1.15 (0.98, 1.34) |
|  | G1.5 | 970,492 | 918 | 0.88 (0.82, 0.94) | 158 | 0.89 (0.76, 1.04) | 88 | 0.86 (0.70, 1.07) | 150 | 0.96 (0.81, 1.12) | 44 | 2.20 (1.62, 2.99) | 20 | 0.93 (0.59, 1.44) |
|  | G2 | 2,471,341 | 1756 | 1.02 (0.97, 1.07) | 308 | 1.06 (0.95, 1.19) | 170 | 0.97 (0.83, 1.12) | 389 | 1.21 (1.09, 1.34) | 43 | 1.23 (0.90, 1.67) | 58 | 1.28 (0.99, 1.67) |
| **Central and Eastern Europe** | G1 | 2,802,347 | 6,798 | 0.87 (0.84, 0.89) | 717 | 0.82 (0.76, 0.88) | 387 | 0.81 (0.73, 0.90) | 676 | 0.96 (0.89, 1.04) | 178 | 1.97 (1.68, 2.31) | 150 | 1.37 (1.15, 1.63) |
|  | G1.5 | 643,077 | 490 | 0.85 (0.77, 0.93) | 85 | 0.89 (0.72, 1.11) | 44 | 0.81 (0.60, 1.09) | 72 | 0.84 (0.67, 1.06) | 30 | 2.74 (1.89, 3.99) | 15 | 1.13 (0.68, 1.88) |
|  | G2 | 1,168,137 | 786 | 1.02 (0.95, 1.09) | 135 | 1.02 (0.86, 1.21) | 82 | 1.03 (0.83, 1.28) | 159 | 1.11 (0.95, 1.30) | 20 | 1.26 (0.80, 1.98) | 23 | 1.13 (0.74, 1.71) |
| Poland | G1 | 960,206 | 1,937 | 0.92 (0.87, 0.98) | 247 | 0.96 (0.85, 1.09) | 126 | 0.92 (0.77, 1.09) | 201 | 0.92 (0.80, 1.06) | 45 | 1.69 (1.25, 2.29) | 44 | 1.41 (1.04, 1.90) |
|  | G1.5 | 141,619 | 52 | 0.81 (0.62, 1.06) | 11 | 1.08 (0.59, 1.94) | 7 | 1.14 (0.54, 2.39) | 12 | 1.04 (0.59, 1.83) | 1 | - | 2 | 0.84 (0.21, 3.38) |
|  | G2 | 240,175 | 148 | 1.02 (0.87, 1.20) | 25 | 0.99 (0.67, 1.46) | 16 | 1.03 (0.62, 1.71) | 31 | 1.19 (0.84, 1.70) | 3 | - | 5 | 1.34 (0.56, 3.22) |
| Former Yugoslavian | G1 | 717,432 | 1,628 | 0.79 (0.74, 0.83) | 210 | 0.68 (0.59, 0.77) | 100 | 0.60 (0.49, 0.74) | 231 | 1.02 (0.89, 1.16) | 68 | 2.16 (1.69, 2.76) | 37 | 1.23 (0.88, 1.72) |
|  | G1.5 | 289,812 | 170 | 0.98 (0.84, 1.14) | 41 | 1.28 (0.95, 1.75) | 12 | 0.74 (0.42, 1.31) | 26 | 0.84 (0.57, 1.24) | 9 | 2.74 (1.42, 5.29) | 6 | 1.05 (0.47, 2.34) |
|  | G2 | 340, 037 | 65 | 1.02 (0.79, 1.30) | 4 | 0.59 (0.22, 1.58) | 5 | 0.89 (0.37, 2.14) | 15 | 1.09 (0.66, 1.82) | 4 | 2.54 (0.82, 7.94) | 5 | 1.29 (0.54, 3.13) |
| Former Soviet Union | G1 | 121,635 | 444 | 0.80 (0.68, 0.94) | 22 | 0.64 (0.42, 0.97) | 19 | 0.93 (0.59, 1.46) | 24 | 0.77 (0.52, 1.15) | 9 | 2.35 (1.22, 4.53) | 9 | 1.40 (0.69, 2.82) |
|  | G1.5 | 30,509 | 55 | 0.67 (0.50, 0.88) | 9 | 0.65 (0.34, 1.26) | 4 | 0.51 (0.19, 1.37) | 5 | 0.50 (0.21, 1.21) | 4 | 2.77 (1.04, 7.41) | 0 | - |
|  | G2 | 87,647 | 140 | 0.99 (0.84, 1.16) | 34 | 1.29 (0.93, 1.81) | 14 | 0.93 (0.55, 1.57) | 26 | 1.09 (0.74, 1.60) | 1 | - | 0 | - |
| Former Czechoslovakia | G1 | 90,051 | 393 | 0.79 (0.69, 0.92) | 30 | 0.71 (0.48, 1.02) | 20 | 0.99 (0.64, 1.54) | 31 | 1.04 (0.73, 1.48) | 6 | 1.34 (0.56, 3.24) | 3 | 0.68 (0.21, 2.04) |
|  | G1.5 | 28,889 | 48 | 0.87 (0.65, 1.17) | 5 | 0.55 (0.23, 1.31) | 10 | 1.74 (0.90, 3.34) | 6 | 0.79 (0.35, 1.75) | 4 | 3.08 (0.99, 9.56) | 0 | - |
|  | G2 | 75,293 | 43 | 0.82 (0.60, 1.11) | 3 | - | 5 | 0.99 (0.41, 2.38) | 5 | 0.52 (0.21, 1.23) | 1 | - | 1 | 0.74 (0.10, 5.23) |
| Estonia | G1 | 167,730 | 925 | 1.01 (0.89, 1.13) | 44 | 0.97 (0.72, 1.31) | 23 | 0.74 (0.48, 1.13) | 35 | 0.94 (0.67, 1.31) | 15 | 2.92 (1.76, 4.87) | 18 | 1.88 (1.17, 3.04) |
|  | G1.5 | 22,473 | 90 | 0.79 (0.61, 1.01) | 11 | 0.82 (0.45, 1.47) | 4 | 0.46 (0.17, 1.23) | 5 | 0.46 (0.19, 1.12) | 8 | 5.19 (2.58, 10.40) | 4 | 4.13 (1.54, 11.03) |
|  | G2 | 215,540 | 294 | 1.08 (0.96, 1.21) | 50 | 0.99 (0.75, 1.31) | 34 | 1.19 (0.85, 1.68) | 59 | 1.19 (0.92, 1.54) | 11 | 2.08 (1.15, 3.76) | 7 | 1.25 (0.59, 2.63) |
| **Rest of Europe** | G1 | 1,783,746 | 4,358 | 0.79 (0.75, 0.82) | 345 | 0.70 (0.63, 0.78) | 253 | 0.87 (0.76, 0.98) | 377 | 0.93 (0.84, 1.03) | 89 | 1.65 (1.32, 2.05) | 53 | 0.78 (0.59, 1.04) |
|  | G1.5 | 327,415 | 426 | 0.91 (0.83, 1.00) | 73 | 0.88 (0.69, 1.10) | 44 | 0.92 (0.68, 1.24) | 78 | 1.10 (0.88, 1.37) | 14 | 1.58 (0.93, 2.66) | 5 | 0.61 (0.25, 1.46) |
|  | G2 | 1,303,204 | 970 | 1.03 (0.96, 1.09) | 173 | 1.10 (0.94, 1.28) | 88 | 0.91 (0.74, 1.12) | 230 | 1.29 (1.13, 1.47) | 23 | 1.20 (0.79, 1.83) | 35 | 1.41 (1.01, 1.98) |
| Germany | G1 | 601,728 | 2,663 | 0.86 (0.81, 0.91) | 209 | 0.86 (0.75, 0.99) | 144 | 0.96 (0.81, 1.13) | 171 | 0.89 (0.76, 1.03) | 44 | 1.58 (1.16, 2.16) | 26 | 0.85 (0.57, 1.26) |
|  | G1.5 | 118,505 | 237 | 0.85 (0.75, 0.97) | 40 | 0.81 (0.59, 1.10) | 31 | 1.09 (0.75, 1.55) | 40 | 1.04 (0.76, 1.41) | 4 | 0.77 (0.28, 2.06) | 2 | 0.51 (0.13, 2.05) |
|  | G2 | 679,787 | 629 | 1.03 (0.95, 1.11) | 113 | 1.08 (0.90, 1.31) | 55 | 0.87 (0.66, 1.13) | 152 | 1.33 (1.14, 1.57) | 15 | 1.18 (0.70, 2.01) | 22 | 1.51 (0.99, 2.30) |
| **NON-WESTERN COUNTRIES** | G1 | 6,298,782 | 4,786 | 0.63 (0.61, 0.65) | 475 | 0.43 (0.39, 0.48) | 339 | 0.56 (0.50, 0.63) | 824 | 0.81 (0.75, 0.87) | 213 | 1.74 (1.49, 2.03) | 133 | 0.75 (0.62, 0.91) |
|  | G1.5 | 1,665,705 | 291 | 0.93 (0.82, 1.04) | 22 | 0.63 (0.41, 0.96) | 37 | 1.42 (1.02, 1.96) | 65 | 1.08 (0.85, 1.39) | 21 | 3.69 (2.36, 5.77) | 9 | 0.44 (0.23, 0.85) |
|  | G2 | 985,479 | 119 | 0.93 (0.77, 1.11) | 6 | 0.46 (0.21, 1.02) | 11 | 0.96 (0.53, 1.73) | 27 | 1.19 (0.81, 1.73) | 6 | 2.53 (1.12, 5.68) | 6 | 0.86 (0.38, 1.94) |
| **South America** | G1 | 626,109 | 740 | 0.57 (0.52, 0.62) | 88 | 0.51 (0.42, 0.63) | 48 | 0.52 (0.39, 0.69) | 81 | 0.56 (0.45, 0.69) | 35 | 1.91 (1.35, 2.70) | 24 | 0.97 (0.63, 1.49) |
|  | G1.5 | 272,829 | 57 | 0.89 (0.69, 1.16) | 7 | 0.98 (0.47, 2.06) | 5 | 0.94 (0.39, 2.27) | 9 | 0.71 (0.37, 1.36) | 6 | 5.29 (2.35, 11.89) | 3 | 0.71 (0.23, 2.21) |
|  | G2 | 157,934 | 19 | 0.86 (0.55, 1.34) | 2 | - | 1 | - | 3 | - | 1 | - | 0 | - |
| **Middle East** | G1 | 2,806,551 | 2,372 | 0.62 (0.59, 0.65) | 205 | 0.38 (0.32, 0.43) | 179 | 0.59 (0.50, 0.69) | 481 | 0.97 (0.88, 1.07) | 93 | 1.57 (1.26, 1.95) | 34 | 0.39 (0.27, 0.57) |
|  | G1.5 | 714,438 | 130 | 0.94 (0.79, 1.12) | 8 | 0.49 (0.24, 0.97) | 18 | 1.55 (0.97, 2.47) | 31 | 1.18 (0.83, 1.69) | 9 | 3.53 (1.81, 6.87) | 2 | 0.24 (0.06, 0.95) |
|  | G2 | 443,344 | 31 | 0.78 (0.54, 1.34) | 0 | - | 1 | - | 10 | 1.61 (0.86, 2.99) | 1 | - | 2 | 0.73 (0.18, 2.92) |
| **Asia** | G1 | 1,768,922 | 1,063 | 0.66 (0.62, 0.70) | 132 | 0.56 (0.47, 0.66) | 74 | 0.57 (0.45, 0.72) | 139 | 0.56 (0.47, 0.67) | 69 | 2.39 (1.85, 3.09) | 55 | 1.29 (0.97, 1.69) |
|  | G1.5 | 428,849 | 58 | 0.79 (0.61, 1.02) | 2 | - | 6 | 1.00 (0.45, 2.23) | 14 | 1.00 (0.59, 1.69) | 4 | 3.08 (1.14, 8.29) | 3 | 0.58 (0.18, 1.81) |
|  | G2 | 185,285 | 48 | 1.24 (0.93, 1.65) | 3 | 0.63 (0.20, 1.94) | 8 | 2.13 (1.06, 4.26) | 9 | 1.25 (0.65, 2.41) | 2 | 2.26 (1.78, 2.89) | 1 | 0.68 (0.09, 4.87) |
| **Africa** | G1 | 1,092,198 | 611 | 0.69 (0.63, 0.76) | 50 | 0.37 (0.29, 0.49) | 38 | 0.52 (0.38, 0.72) | 123 | 0.92 (0.77, 1.10) | 16 | 1.01 (0.61, 1.66) | 20 | 0.75 (0.47, 1.17) |
|  | G1.5 | 249,588 | 46 | 1.18 (0.88, 1.58) | 5 | 1.19 (0.49, 2.88) | 8 | 2.51 (1.25, 5.03) | 11 | 1.56 (0.86, 2.82) | 2 | - | 1 | 0.39 (0.06, 2.75) |
|  | G2 | 198,915 | 21 | 0.74 (0.48, 1.14) | 1 | - | 1 | - | 5 | 0.93 (0.38, 2.23) | 2 | - | 3 | 1.97 (0.63, 6.15) |
| **REST OF THE WORLD** | G1 | 182,696 | 159 | 0.59 (0.49, 0.72) | 13 | 0.42 (0.24, 0.72) | 11 | 0.62 (0.34, 1.19) | 21 | 0.65 (0.42, 1.01) | 6 | 1.73 (0.78, 3.87) | 5 | 1.03 (0.43, 2.47) |
|  | G1.5 | 43,152 | 7 | 0.40 (0.16, 0.96) | 2 | - | 0 | - | 0 | 0 | 0 | - | 0 | - |
|  | G2 | 86,971 | 203 | 1.07 (0.93, 1.23) | 37 | 1.06 (0.76, 1.46) | 21 | 1.05 (0.69, 1.61) | 32 | 1.24 (0.87, 1.77) | 3 | 0.81 (0.26, 2.52) | 3 | 0.44 (0.06, 3.14) |

HRs adjusted for age, educational status, disposable income, marital status, and calendar years – all characteristics measured at exit date of the study
